# Targeting leucine-rich alpha-2-glycoprotein-1 (LRG1) and the LRG1-cytochrome *c*-Apaf-1 axis in cancer therapy

**DOI:** 10.18632/oncoscience.667

**Published:** 2026-07-29

**Authors:** Ronald Jemmerson

**Affiliations:** ^1^Department of Microbiology and Immunology, University of Minnesota, Minneapolis, MN 55455, USA

**Keywords:** leucine-rich alpha-2-glycoprotein-1, cytochrome *c*, apoptotic protease activating factor-1, targeted degradation, antibody therapy

## Abstract

Leucine-rich alpha-2-glycoprotein-1 (LRG1) has received much attention as a prognostic indicator in cancer therapy where high serum levels generally predict a poor outcome. Recently, it has been shown to play an active role in cancer progression and is being pursued as a novel target in cancer therapy. LRG1 acts in neovascularization of tumors resulting in weakened blood vessels and ineffective delivery of chemotherapeutic drugs. A monoclonal antibody (mAb) against LRG1 has shown efficacy in blocking this function of LRG1, normalizing the vasculature, and improving drug delivery in mice. Extracellular LRG1 is also anti-apoptotic and promotes cell proliferation and metastasis, all through epidermal growth factor receptor family signaling. In mice, anti-LRG1 antibody therapy has been shown to inhibit these effects of extracellular LRG1 and to enhance the anti-cancer effect of immune checkpoint blockade (ICB) therapy in mice. Pre-clinical testing of the humanized version of the LRG1 mAb is underway in the United Kingdom. Intracellular LRG1 has been reported to inhibit apoptosis by blocking the binding of Cyt *c* to apoptotic protease activating factor1 (Apaf-1) and to have other cytoplasmic functions. A nanoscale proteolysis targeting chimera (nano-PROTAC) with improved cytoplasmic delivery has been developed which enhanced apoptosis in tumors of mice. Targeting LRG1 in the aberrant angiogenesis of endothelial cells and the LRG1-Cyt *c*-Apaf-1 axis in cancer cells, along with other putative intracellular interactions of LRG1, show potential as supplementary, if not alternative, approaches to cancer therapy.

## INTRODUCTION

Increased leucine-rich alpha-2-glycoprotein-1 (LRG1) in blood and cancer cells has become a hallmark of many cancers and a prognostic indicator predicting poor outcomes when levels of LRG1 are elevated [1, 2]. LRG1 also plays an active role in cancer progression [1, 2]. Recent attention has been directed toward inhibiting the functions of LRG1 as an approach to cancer therapy [3, 4]. LRG1, a 50 kDa fully glycosylated protein in blood and 45 kDa partially glycosylated protein in cancer cells, is the founding member of the large set of proteins with leucine-rich repeats that generally assume a horseshoe shape [5, 6]. Interactions with other proteins usually occur along the concave surface which provides a platform for protein-protein interactions [7].

In LRG1, this surface has a net negative charge suitable for interaction with positively charged proteins [6]. In 2002, transforming growth factor-β1 (TGF-β1), a slightly basic protein dimer (25 kDa), was the first LRG1 ligand identified in a study in which LRG1 was found to be a marker for high endothelial venules [8]. Six years later serum LRG1 was found to bind the highly basic protein, cytochrome *c* (Cyt *c*; 12.5 kDa) when it was discovered as the serum component blocking detection of extracellular Cyt *c* in an enzyme-linked immunosorbent assay [9]. Other LRG1 ligands that have been reported recently include the nuclear factor kappa-light-chain-enhancer of activated B cells (NFκB) subunit 1 and fibronectin 1 [10, 11]. Several cell-surface receptors have been identified as LRG1 binding partners including endoglin, latrophilin-2, and at least two members of the epidermal growth factor receptor family, EGFR (epidermal growth factor receptor) and human epidermal growth factor receptor 3 (HER3) [6, 12–14]. The net effect of extracellular LRG1 signaling likely results from the ligands bound and the receptors that are engaged.

Except for the canonical TGF-β1 signaling pathway that leads to aberrant neovascularization (discussed below), our understanding of the pathways resulting in other effects of LRG1 is incomplete [12]. LRG1 knockout mice develop normally and display no overt abnormalities, although deficiency in the Th17 lymphocyte population has been observed [1, 15]. The normal role of LRG1 may be to promote cell survival in the maintenance of homeostasis under stress conditions [16].

## ANTI-APOPTOTIC, PROLIFERATIVE, AND MIGRATORY EFFECTS OF EXTRACELLULAR LRG1

In 2010 the first *in vitro* biological function of LRG1 was reported. LRG1 was observed to delay the onset of apoptosis in cultured human and mouse lymphocytes treated with extracellular Cyt *c* [17]. Apoptosis was defined by the phenotype of the cell, i.e., the exposure of phosphatidylserine on the cell surface [18]. The very low molar ratio of LRG1 to Cyt *c* providing maximal protection from apoptosis (1:100) suggested that the LRG1-Cyt *c* complex may deliver a survival signal as opposed to LRG1 simply blocking the extracellular pro-apoptotic effect of Cyt *c*. However, this remains to be directly demonstrated. The LRG1-Cyt *c* interaction appears to be physiological as complexes of the two proteins have been observed in the blood of mice where LRG1 interfered with the production of pro-inflammatory cytokines by macrophages *in vitro* in response to extracellular Cyt *c* [19]. In another study, injection of LRG1 at the site of fat grafts in mice was shown to inhibit the onset of apoptosis of adipocytes in the graft, thus confirming a function of LRG1 in cell survival [20]. Any role for Cyt *c* in the survival effect observed was not examined in that study.

Early evidence that LRG1 is elevated in the blood of cancer patients appeared in 2007 with even more evidence accumulating 3–5 years later [21–24]. The first reports that LRG1 plays a role in cancer cell survival, proliferation, and metastasis did not emerge until 2015 [25]. Knockdown or overexpression of LRG1 in human glioblastoma cells employing transfection techniques and transplantation of the cells as xenografts in mice showed that the proliferation and migration of the cells were dependent on LRG1. This effect involved LRG1 secreted by the glioblastoma cells, as the cell membrane-associated TGF-β signaling pathway was implicated employing a specific inhibitor [26].

The same research group showed that knockdown of LRG1 resulted in a decrease in tumor weight by more than half with decreased expression of cyclins D1, B, and E, indicating a blockage in cell cycle progression [26]. An increase in apoptosis in LRG1 knockdown cells was demonstrated phenotypically by flow cytometry as well as by altered expression of proteins involved in apoptosis, including a 2- to 3-fold decrease in Bcl-2 with a more than 2-fold increase in both Bax and cleaved caspase-3. Bcl-2 plays a role in blocking the release of Cyt *c* from mitochondria and Bax facilitates Cyt *c* release [27–30]. In the cytoplasm Cyt *c* binds to Apaf-1 leading to activation of caspase-9, which then cleaves caspase-3 [31, 32]. Multiple research groups have since demonstrated a pro-survival effect of LRG1 on several types of cancer cells. Their studies have generally shown increased expression of the anti-apoptotic protein Bcl-2 and decreased expression of the pro-apoptotic protein Bax [4, 10, 33–35].

**Figure 1:**
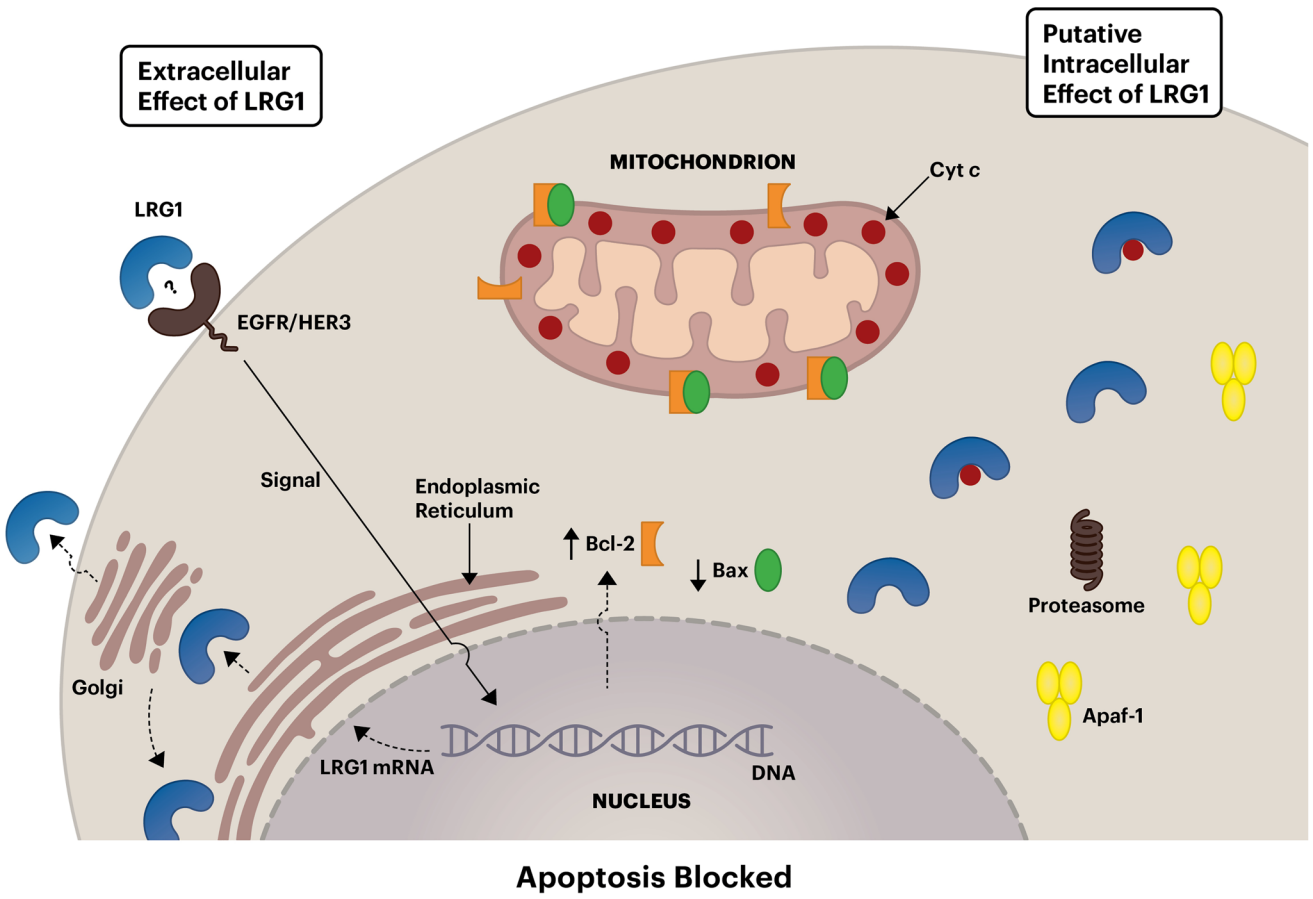
Schematic cartoon of the LRG1-Cyt *c*-Apaf-1 axis in cancer cells. Cells overexpressing LRG1 secrete the glycoprotein which can then bind to receptors of the EGFR family on the cell surface. LRG1 is also in circulation. Accessory ligands, if any, have not been defined in the signaling by extracellular LRG1 (indicated by “?”). Signaling through the receptors to the nucleus affects gene transcription, elevating the level of anti-apoptotic Bcl-2 and lowering pro-apoptotic Bax in the cytoplasm (dotted lines from the nucleus indicate translocation of mRNA to the cytoplasm and translation to protein). Bcl-2 and Bax proteins translocate to the outer mitochondrial membrane and impact the release of Cyt *c*. In this scenario, Bcl-2 associates with Bax and blocks Cyt *c* release. However, some Cyt *c* molecules may escape mitochondria in live cells and become trapped in the cytoplasm by intracellular LRG1. By preventing Apaf-1 from binding cytoplasmic Cyt *c* apoptosis is blocked.

With the confirmation from several research groups that a key anti-apoptotic effect of LRG1 is to impact the release of Cyt *c* from mitochondria, it seems appropriate to consider an extended pathway or axis beginning with LRG1 binding to a receptor on a cell, through regulating expression of genes blocking Cyt *c* release from mitochondria, and, ultimately, inhibiting the activation of Apaf-1 and apoptosis. This axis is represented as a cartoon in Figure 1. LRG1 expression is increased in circulation during infections, inflammation, and tissue damage largely in response to cytokines that are produced by a variety of cells including hepatocytes [36]. When LRG1 is overexpressed in cells, it is secreted as a fully glycosylated protein and acts in an autocrine manner [37]. Included in the cartoon scheme in Figure 1 is binding of LRG1 to a few molecules of Cyt *c* in the cytoplasm that may have been released from “faulty” mitochondria in the absence of an apoptotic insult. In MCF-7 breast cancer cells overexpressing LRG1, Cyt *c* was present in the cytoplasm without inducing apoptosis, however it was bound to LRG1 [37].

The studies of LRG1 in cancer discussed above have also shown that, in addition to a role in cell survival and proliferation, LRG1 plays an active role in cancer cell migration and invasion [1, 2, 10, 11, 13]. Increased LRG1 has been observed in the serum of patients with lung and melanoma cell metastases. In two independent studies employing mouse models of these cancers, deletion of the LRG1 gene in the mice reduced metastases [38, 39]. A mAb specific for LRG1 was tested in the lung cancer study and found to slow metastatic growth [38]. In the melanoma study, metastasis was dependent on EGFR signaling, similar to receptor engagement leading to the antiapoptotic effect of LRG1 [39].

## ANTI-LRG1 NEUTRALIZING ANTIBODIES BLOCK ABERRANT VASCULARIZATION IN TUMORS ENHANCING DRUG DELIVERY AND DIRECTLY INHIBIT SURVIVAL AND METASTATIC SIGNALS TO CANCER CELLS

**Figure 2:**
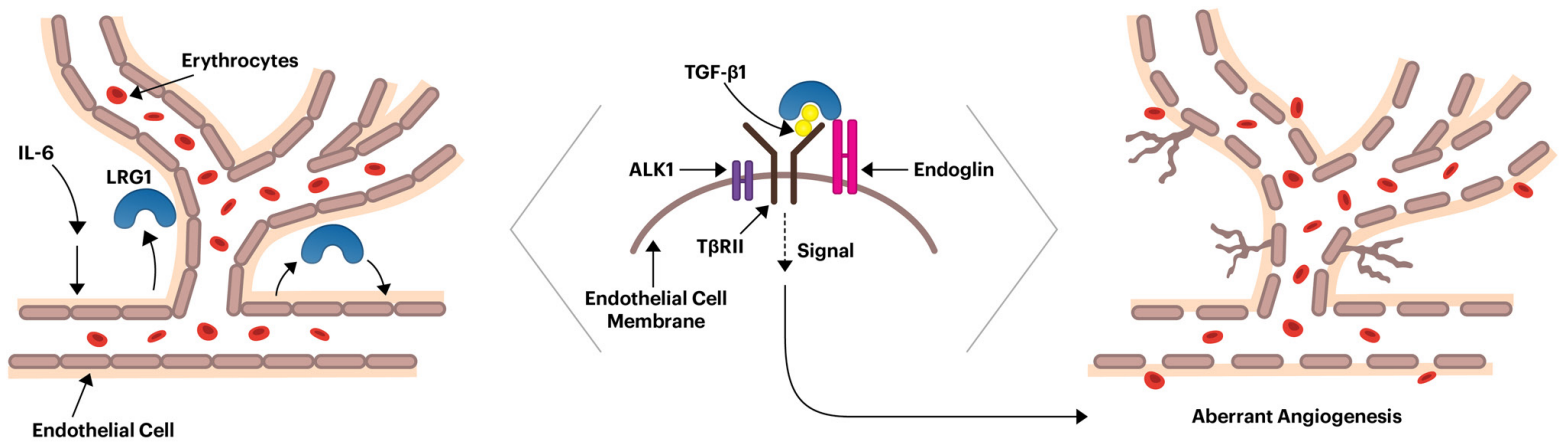
LRG1 induces aberrant angiogenesis (neovascularization). IL-6 is a prominent cytokine produced by a variety of cells in inflammation that acts on endothelial cells to secrete LRG1 [43]. LRG1 is also a circulating protein produced by hepatocytes and neutrophils, primarily. LRG1 interacts with endoglin and TGF-β1 to activate TβIIR, in association with the kinase, ALK1. The blood vessels formed following signaling through this pathway are leaky and weakened.

In 2013, LRG1 was shown to play a key role in eye disease by affecting aberrant neovascularization during tissue repair in that the resulting blood vessels are abnormally formed and leaky [12]. In that study extracellular LRG1 was found to bind endoglin which, in association with TGF-β1 and the TβRII receptor signals endothelial cells through the canonical TGF-β1 pathway involving ALK1 (see Figure 2). Mouse mAbs blocked LRG1 signaling through this pathway and normalized neovascularization in mouse models of eye disease [40]. A mAb with high affinity binding to LRG1 was engineered for use in clinical applications in humans and further optimized to maintain its affinity [41]. Study of the mAb, magacizumab and its derivatives is in the pre-clinical testing phase in the United Kingdom [42].

In addition to its application in eye disease, the mouse precursor of magacizumab (15C4 mAb) has been shown to block aberrant vascularization in mouse tumors, improve delivery of a chemotherapeutic drug, and enhance the immune response within the tumors [3]. Mouse melanoma and lung cancer cells were transplanted into mice and the effects of the 15C4 mAb on tumor growth with and without cisplatin treatment were examined. The mAb alone improved vascular function within the tumor, slowed tumor growth, and enhanced mouse survival, while in conjunction with cisplatin the effects of chemotherapy were improved. Moreover, the mAb increased the infiltration of CD8^+^ T cells into the tumor, enhancing immune checkpoint blockade (ICB) by anti-PD-1 antibody treatment [3].

Other researchers provided evidence that LRG1 is increased in melanoma recurrence in humans following ICB treatment suggesting that targeting LRG1 could be useful as a supplementary therapy in cases where ICB has failed [44]. Blocking LRG1 could also supplement the effect of other angiogenic inhibitors such as anti-vascular endothelial growth factor (VEGF) mAbs because LRG1 and VEGF signal through different pathways [3, 45]. Whereas the anti-VEGF mAb, Bevacizumab, inhibits blood vessel formation, the anti-LRG1 mAb, Magacizumab, acting against the abnormal neovascularization of tumors induced by LRG1, normalizes vascularization allowing for more efficient drug delivery [3, 40, 41].

**Figure 3:**
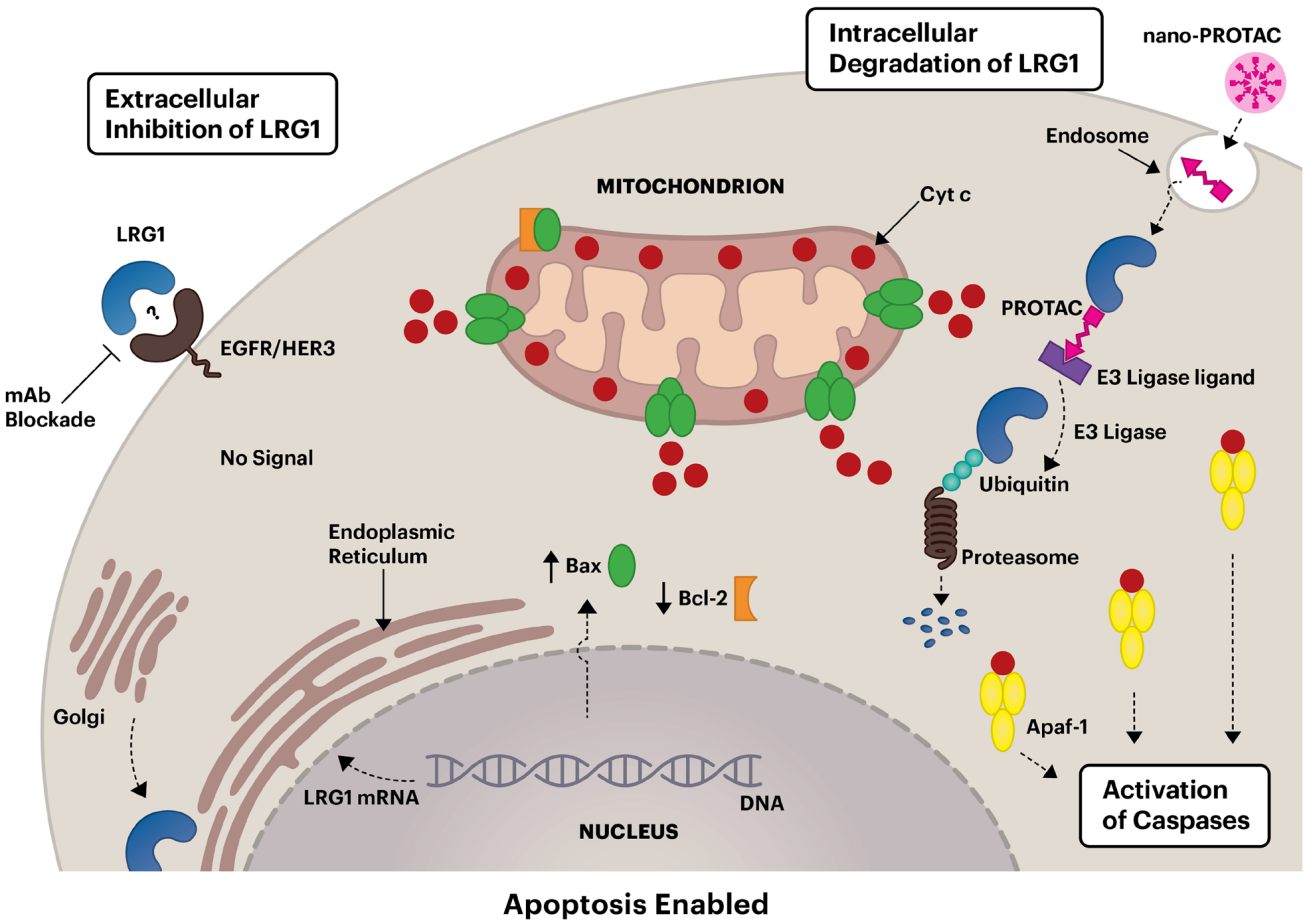
Schematic cartoon of the LRG1-Cyt *c*-Apaf-1 axis in a cell treated with both the anti-LRG1 mAb and a nano-PROTAC to degrade LRG1 in the cytoplasm. The anti-LRG1 mAb, magacizumab blocks signaling through EGFR family members and the cancer cell remains poised to respond to apoptotic insults. Decreased expression of Bcl-2 allows Bax to oligomerize in the mitochondrial outer membrane and facilitate the translocation of Cyt *c* to the cytoplasm. Delivery of the nano-PROTAC into the cytoplasm leads to its association with both LRG1 and the ligand for E3 ubiquitin ligase, which recruits the enzyme for attaching ubiquitin to LRG1. The glycoprotein is then degraded in the proteasome. In the absence of competition from cytoplasmic LRG1, Apaf-1 binds Cyt *c* that is released from mitochondria into the cytoplasm and initiates caspase activation, culminating in apoptosis.

In a recent study, another anti-LRG1 mAb (C4) was used to inhibit proliferation of multiple myeloma cells in mice in response to LRG1 [34]. A decrease in Bcl-2 expression and an increase in Bax were observed in response to mAb C4 treatment and apoptosis was enabled. In that study, LRG1 present in platelet-derived exosomes was key to myeloma cell growth. Exosomes are membrane-bound vesicles released from activated platelets and cells. They contain lipids, proteins, and nucleic acids [46]. The exosomes fuse with the cell membrane or are endocytosed allowing entry of the contents into the cytoplasm of the recipient cells. These molecules act as messengers signaling various pathways and altering the phenotype of the recipient cells. In the myeloma study, inhibition by mAb C4 suggests that the proliferative effect of exosomal LRG1 was induced at the cell surface. In other studies, exosomal LRG1 derived from non-small cell lung cancer cells had both an extracellular effect promoting angiogenesis by signaling through the TGF-β pathway and, reportedly, an intracellular effect of promoting growth and metastasis by binding the transcription factor, NFκB (subunit 1), and its enhancer, fibronectin 1 [10, 11, 47]. The mAb treatment would not impact these or other potential intracellular functions of LRG1. See Figure 3, left side for a schematic cartoon showing the effect of anti-LRG1 mAb treatment on apoptosis. Unable to enter the cell, the mAb has no effect on cytoplasmic functions of LRG1 other than those activated through the EGFR family of receptors.

## TARGETING LRG1 IN THE PUTATIVE INTRACELLULAR ARM OF THE LRG1-CYT *C*-APAF-1 AXIS AND OTHER CYTOPLASMIC INTERACTIONS OF LRG1

LRG1, like the apoptotic protease activating factor (Apaf-1), is inhibited in binding Cyt *c* by tri-methylation of lysine at Cyt *c* residue 72 and competes with Apaf-1 for binding Cyt *c* [17]. The finding that partially glycosylated LRG1 (45 kDa) is retained in the cytoplasm of cells raised the possibility that LRG1 may compete with Apaf-1 for binding Cyt *c* physiologically and, thus, inhibit apoptosis [37]. This possibility was confirmed in one study of MCF-7 breast cancer cells. Notably, Cyt *c* was present in the cytoplasm of cells overexpressing LRG1 by gene transfection, but it was bound to LRG1 and not to Apaf-1, thus promoting cell survival. In apoptotic cells where LRG1 had been degraded, especially shown in LRG1 knockdown cells, Cyt *c* was preferentially bound to Apaf-1 [37]. The interaction of Cyt *c* with Apaf-1 was discovered 30 years ago and quickly confirmed [31, 32, 48]. The intracellular interaction of LRG1 with Cyt *c* was reported 5 years ago and has not yet been independently validated, nor has it been shown to occur in types of cancer cells other than the breast cancer cell line, MCF-7 [37]. Herein this interaction within cells will be described as “putative.” The affinity of LRG1 for Cyt *c* (K_d_ = 1.58 × 10^−13^ M) is several orders of magnitude greater than the affinity of Apaf-1 for Cyt *c* under physiological conditions (K_d_ = 2.5 × 10^−8^ M), indicating that LRG1 would be an effective Cyt *c* trap in the cytoplasm blocking the activation of Apaf-1 [49, 50].

As a distinct approach from the use of mAbs to block extracellular LRG1 functions, targeting of intracellular LRG1 for degradation could have two effects, reducing the amount of LRG1 that is secreted from cells and removing a block on apoptosis induction via the putative intracellular arm of the LRG1-Cyt *c*-Apaf-1 axis, as well as blocking other reported functions of LRG1 in the cytoplasm.

There are two approaches to targeted degradation. Extracellular and membrane-bound proteins can be directed to the endosomal-lysosomal pathway using lysosome targeting chimeras (LYTACs). The LYTACs act as molecular glues with one end bound to the target protein and the other capable of binding a lysosome-shuttling receptor [51]. Once delivered to the cell by this pathway, the protein-LYTAC cannot enter the cytoplasm, so this would not be an effective way to remove cytoplasmic LRG1. The other approach to targeted degradation is appropriate for cytoplasmic proteins. In this approach the molecular glue is a proteolysis-targeting chimera (PROTAC) that is constructed to contain a protein-binding domain at one end with a linker attached to a ligand of E3 ubiquitin ligase at the other end [52]. Simultaneous PROTAC binding to the protein being targeted and the ligase via its ligand allows the ligase to attach ubiquitin to the protein, thus directing the complex to the 26s proteasome for degradation. (See Figure 3, right side.)

Recently, directed degradation of intracellular LRG1 has been achieved employing a PROTAC [4, 53]. The protein-binding domain employed was a 12-amino acid-long peptide (ET), identified to bind LRG1 using phage display technology. When labeled with the fluorescent tag, Cy5, ET localized preferentially to mouse mammary tumors and metastases *in vivo* [4]. For targeted degradation of LRG1, the peptide was covalently attached to lenalidomide, a ligand of E3 ubiquitin ligase, employing a short peptide linker [53]. Subsequently, to overcome trapping of the PROTAC in the endosome and improve delivery to the cytosol, a lipid nanoparticle, a nanoscale PROTAC (nano-PROTAC), was created containing both ET and lenalidomide, each covalently attached to polyethylene glycol and a tertiary amine, ionizable in the acidic environment of the endosome [4].

The ionizable nano-PROTAC was superior to the non-ionizable form by decreasing mammary tumor weight in mice more than two-fold in comparison, consistent with improved cytoplasmic delivery of the PROTAC. Among biochemical parameters that were quantified, the 45 kDa form of LRG1 was decreased by more than half in the tumor cells following injection with the ionizable nano-PROTAC. A similar reduction was observed in Bcl-2 and an increase in Bax, with a two-fold increase in cleaved caspase 3. Phosphorylated protein kinase B (also known as AKT) was significantly diminished, consistent with reduced signaling through EGFR [54].

In several preliminary experiments employing a mouse breast cancer cell line (4T1) to optimize lipid carbon chain length, nano-PROTAC concentration, and treatment time, reduction in intracellular LRG1 expression was consistently observed. Furthermore, the decrease in LRG1 in the mammary tumor cells in this study is consistent with a significant decrease reported by the same investigators in kidney tissues in a mouse model of fibrosis where the naked PROTAC (not contained in the lipid nanoparticle) was employed [53].

Altered expression of the apoptosis-related genes in the mammary tumor study was apparently due to decreased secretion of LRG1 and reduced signaling through cell surface receptors. In the earlier study in which LRG1 was targeted in kidney fibrosis, the PROTAC lowered the amount of both intracellular and extracellular LRG1 in a human kidney epithelial cell line *in vitro* [53]. Reduced intracellular LRG1 should also have lowered competition with Apaf-1 for binding Cyt *c*, thus making the cells more susceptible to apoptosis, and inhibited other functions related to metastasis such as binding of LRG1 to NFκB subunit 1 and fibronectin 1. However, these functions were not examined in these studies [4, 53].

In summary, the impacts of targeting extracellular LRG1 employing a neutralizing mAb and intracellular LRG1 by targeted proteasomal degradation are represented in the cartoon in Figure 3. In the absence of the LRG1 signal through its cell-surface receptor, the level of Bcl-2 is decreased relative to the level of Bax, allowing Bax to oligomerize in the mitochondrial outer membrane, thus enabling the release of Cyt *c* to the cytoplasm. Degradation of cytoplasmic LRG1 would remove the putative trap for Cyt *c* that would prevent its binding Apaf-1, leading to the activation of caspases and, ultimately, to apoptosis.

## BENEFITS AND POTENTIAL LIMITATIONS TO THESE APPROACHES

LRG1 has both extracellular and putative intracellular effects promoting cell survival, proliferation, and metastasis, thus representing a unique target for cancer therapy.

Blockade of extracellular LRG1 by a mAb and targeted degradation of intracellular LRG1 together could provide a one-two punch to block its effects on cancer progression.

Over 40 mAbs have been approved for clinical use in cancer therapy by the United States Food and Drug Administration [55]. In contrast, no PROTACs have yet been approved, although over 30 are in various stages of clinical trials, mostly for cancer [56]. Thus, there is a wealth of knowledge which should facilitate optimizing clinical targeting of extracellular LRG1 employing a mAb and targeting of intracellular LRG1 employing a PROTAC.

The anti-LRG1 mAb, magacizumab by itself has several distinct effects: countering the inhibition of apoptosis, blocking proliferation and the promotion of metastases, and normalizing tumor vasculature [1, 3, 40, 41]. The effect of LRG1 resulting in weakened blood vessels involves a signaling pathway distinct from the VEGF pathway that is blocked by angiogenesis inhibitors such as the mAb, bevacizumab [12, 45]. Magacizumab may offer a supplementary therapy making angiogenesis blockade more effective [3, 40, 41]. Likewise, in mice, anti-LRG1 mAb treatment has been shown to supplement ICB therapy, thus providing hope toward enhancing the application of this immunotherapeutic approach to cancer treatment [3].

LRG1 is not required for normal development and its absence in LRG1 gene-targeted knock-out mice does not confound the health of mice maintained in specific pathogen-free environments [1]. As an acute-phase protein its physiological role may be to promote survival of cells under stress conditions, such as tissue injury and infection [16]. In cancer patients who are infected with a pathogen, it is possible that off-target effects of mAb therapy could occur. By decreasing circulating LRG1, both the mAb and nano-PROTAC therapies could complicate patient recovery from diseases unrelated to cancer. Neither the anti-LRG1 mAb nor nano-PROTAC have shown signs of off-target effects in mouse experiments [1, 4, 40, 53].

The high concentration of LRG1 in the blood, in the micrograms-per-milliliter range and elevated in many cases of cancer, could prevent delivery of the mAb to the microenvironment of the cancer cells where LRG1 binds its receptor [1]. Some other soluble ligands that are frequently targeted by mAbs in cancer therapy, such as VEGF and interleukin-2, are present in the blood in the picograms-per-milliliter range [57, 58]. Higher concentrations of LRG1 in tumors relative to blood may suggest preferential targeting of the mAb to tumors [59]. The successful experiments in mice support this possibility. Similarly, circulating LRG1 could hinder delivery or processing of the nano-PROTAC to cancer cells. However, in mice, fluorescent Cy5-nano-PROTAC preferentially localized in mammary tumors, even slightly more effectively than in the liver, which is the major producer of LRG1 in the body, and significantly more efficiently than a nano-PROTAC containing a non-targeting peptide [4].

Degradation of LRG1 in the cytoplasm alone may not make Apaf-1 susceptible to activation as there are redundant mechanisms for blocking the interaction between Cyt *c* and Apaf-1. For example, heat shock protein 27 and nucleotides have been shown separately to bind Cyt *c* and inhibit its interaction with Apaf-1 [60, 61]. Also, phosphorylation of Apaf-1 allows binding of the protein 14-3-3 epsilon to Apaf-1 which inhibits its association with Cyt *c* [62].

LRG1 has been implicated as a survival, proliferative, and metastatic factor in 18 types of cancer [63]. However, there are some reports that LRG1 has the opposite effect in cancer [64, 65]. The basis for this paradox may be the involvement of an alternative signaling pathway. While EGFR family members have been implicated in the pro-cancerous functions of LRG1, TβRI (distinct from TβRII, that is involved in neovascularization) has been implicated in the anti-cancerous effects of LRG1 in studies of Lewis lung carcinoma and esophageal squamous cell carcinoma [64, 65]. Levels of LRG1 and its mRNA were low in the cancer cells, suggesting that the biochemical pathways within the cells, perhaps owing to differential gene expression, were different from cells expressing elevated levels of LRG1. In patients with cancer characterized by low expression of LRG1 and where LRG1 is pro-apoptotic, perhaps LRG1 itself could be useful in therapy.

## CONCLUSIONS

Induction of apoptosis via activation of Apaf-1 by Cyt *c* is a pathway contributing to effective cancer therapy for many drugs. LRG1 appears to interfere with the activation of Apaf-1 in more than one way. As an extracellular signaling molecule, LRG1 affects the expression of genes that regulate the release of Cyt *c* from mitochondria, decreasing pro-apoptotic Bax and increasing anti-apoptotic Bcl-2. Anti-LRG1 mAb therapy reverses the extracellular pro-survival and other pro-cancerous effects of LRG1. Moreover, anti-LRG1 mAb therapy may enhance the efficacy of conventional chemotherapy by improving the tumor vasculature for more effective drug delivery and may enhance ICB treatment as both have been shown in mouse models of cancer.

LRG1 binds the same site on Cyt *c* as Apaf-1 and can compete with Apaf-1 for binding Cyt *c*. Where this may occur in the cytoplasm of cancer cells, a LRG1-targeted nano-PROTAC, optimized for endosomal escape into the cytoplasm, could enable apoptosis more effectively. Other intracellular functions of LRG1 impacting cancer cell growth and metastasis have been reported that could also be targeted in this way. In addition, intracellular degradation leads to a decrease in LRG1 secretion, thus reducing the extracellular survival, proliferation, and migration/invasion effects of LRG1 resulting from signaling through the EGFR family of receptors.

Much more is yet to be learned about the signaling pathways involved in the execution of the effects of LRG1, including involvement of its ligands and receptors, which may lead to additional avenues for therapy. Neither mAb targeting of extracellular LRG1 nor nano-PROTAC targeting of intracellular LRG1 has reached the clinical trial stage, although preliminary results from studies in mice are encouraging and the humanized anti-LRG1 mAb, magacizumab is in pre-clinical testing in the United Kingdom for treatment of a variety of diseases.

## Abbreviations

Apaf-1: apoptotic protease activating factor-1
Cyt *c*: cytochrome *c*
EGFR (HER1): epidermal growth factor receptor
HER3: human epidermal growth factor receptor 3
LRG1: leucine-rich alpha-2-glycoprotein-1
ICB: immune checkpoint blockade
IL-6: interleukin-6
mAb: monoclonal antibody
nano-PROTAC: nanoscale proteolysis targeting chimera
NFκB: nuclear factor kappa-light-chain enhancer of activated B cells
TβRII or I: canonical receptors for TGF-β1
TGF-β1: transforming growth factor-beta 1
VEGF: vascular endothelial growth factor
